# Precocious Metamorphosis in the Juvenile Hormone–Deficient Mutant of the Silkworm, *Bombyx mori*

**DOI:** 10.1371/journal.pgen.1002486

**Published:** 2012-03-08

**Authors:** Takaaki Daimon, Toshinori Kozaki, Ryusuke Niwa, Isao Kobayashi, Kenjiro Furuta, Toshiki Namiki, Keiro Uchino, Yutaka Banno, Susumu Katsuma, Toshiki Tamura, Kazuei Mita, Hideki Sezutsu, Masayoshi Nakayama, Kyo Itoyama, Toru Shimada, Tetsuro Shinoda

**Affiliations:** 1 National Institute of Agrobiological Sciences, Tsukuba, Japan; 2 Department of Agricultural and Environmental Biology, Graduate School of Agricultural and Life Sciences, The University of Tokyo, Tokyo, Japan; 3 Initiative for the Promotion of Young Scientists' Independent Research, Graduate School of Life and Environmental Sciences, University of Tsukuba, Tsukuba, Japan; 4 Institute of Genetic Resources, Faculty of Agriculture, Kyushu University Graduate School, Fukuoka, Japan; 5 Institute of Floricultural Sciences, National Agriculture and Food Research Organization, Tsukuba, Japan; 6 School of Agriculture, Meiji University, Kawasaki, Japan; Janelia Farm Research Campus, Howard Hughes Medical Institute, United States of America

## Abstract

Insect molting and metamorphosis are intricately governed by two hormones, ecdysteroids and juvenile hormones (JHs). JHs prevent precocious metamorphosis and allow the larva to undergo multiple rounds of molting until it attains the proper size for metamorphosis. In the silkworm, *Bombyx mori*, several “moltinism” mutations have been identified that exhibit variations in the number of larval molts; however, none of them have been characterized molecularly. Here we report the identification and characterization of the gene responsible for the *dimolting* (*mod*) mutant that undergoes precocious metamorphosis with fewer larval–larval molts. We show that the *mod* mutation results in complete loss of JHs in the larval hemolymph and that the mutant phenotype can be rescued by topical application of a JH analog. We performed positional cloning of *mod* and found a null mutation in the cytochrome P450 gene *CYP15C1* in the *mod* allele. We also demonstrated that *CYP15C1* is specifically expressed in the corpus allatum, an endocrine organ that synthesizes and secretes JHs. Furthermore, a biochemical experiment showed that CYP15C1 epoxidizes farnesoic acid to JH acid in a highly stereospecific manner. Precocious metamorphosis of *mod* larvae was rescued when the wild-type allele of *CYP15C1* was expressed in transgenic *mod* larvae using the GAL4/UAS system. Our data therefore reveal that *CYP15C1* is the gene responsible for the *mod* mutation and is essential for JH biosynthesis. Remarkably, precocious larval–pupal transition in *mod* larvae does not occur in the first or second instar, suggesting that authentic epoxidized JHs are not essential in very young larvae of *B. mori*. Our identification of a JH–deficient mutant in this model insect will lead to a greater understanding of the molecular basis of the hormonal control of development and metamorphosis.

## Author Summary

The number of larval instars in insects varies greatly across insect taxa and can even vary at the intraspecific level. However, little is known about how the number of larval instars is fixed in each species or modified by the environment. The silkworm, *Bombyx mori*, provides a unique bioresource for investigating this question, as there are several “moltinism” strains that exhibit variations in the number of larval molts. The present study describes the first positional cloning of a moltinism gene. We performed genetic and biochemical analyses on the *dimolting* (*mod*) mutant, which shows precocious metamorphosis with fewer larval–larval molts. We found that *mod* is a juvenile hormone (JH)–deficient mutant that is unable to synthesize JH, a hormone that prevents precocious metamorphosis and allows the larvae to undergo multiple rounds of larval–larval molts. This JH–deficient mutation is the first described to date in any insect species and, therefore, the *mod* strain will serve as a useful model for elucidating the molecular mechanism of JH action. Remarkably, precocious larval–pupal transition in *mod* larvae does not occur in the first or second instar, suggesting that morphostatic action of JH is not necessary for young larvae of *B. mori*.

## Introduction

The number of larval instars in insects varies greatly across insect taxa, and can even vary at the intraspecific level [1], [2], [3]. In general, phylogenetically higher insects tend to have fewer larval instars (three to eight) compared to species from basal lineages, such as Ephemeroptera, Odonata and Plecoptera (more than ten) [1], [2], [3]. In many species, the number of larval instars is affected by genetic and environmental factors, such as temperature, nutritional conditions, photoperiod, humidity, injuries, and sex [1], [2]. The variation in the number of larval instars in the insect lifecycle is generally considered to be an adaptive response to diverse environmental conditions in order to ensure the attainment of a threshold-size for metamorphosis [1], [2], [3], [4].

The silkworm *Bombyx mori*, a classic model organism for endocrinology, has been reared by humans for thousands of years, and more than 1,000 strains are currently maintained [5], [6], [7]. Among these, several “moltinism” strains have been identified that exhibit variations in the number of larval instars [6], [7]. Silkworms typically have five larval instars, but the moltinism strains vary between three and seven [6], [7]. For example, precocious larval-pupal metamorphosis is observed in the *mod* (*dimolting*, chromosome 11–27.4 cM), *rt* (*recessive trimolting*, 7–9.0) and *M^3^* (*Moltinism*, 6–24.1) strains, while extra larval molting is observed in the *M^5^* (*Moltinism*, 6–24.1) strain [6], [7]. To date, however, none of these loci has been characterized at the molecular level. Given the availability of whole genome data and post-genomic tools in *B. mori* [8], [9], [10], these strains offer a valuable resource for elucidating the molecular mechanism that underlies plasticity in the number of larval instars.

Here we report the identification and characterization of the gene responsible for the *mod* mutation that causes precocious larval-pupal metamorphosis in the third or fourth instar [11]. Most *mod* larvae form larval-pupal intermediates, but some individuals can become miniature moths with normal fertility. Thus, the *mod* mutant strain can be maintained as homozygous stocks [6], [11], [12]. We demonstrate that the *mod* locus encodes CYP15C1, a cytochrome P450 involved in the biosynthesis of juvenile hormones (JHs), whose “status quo” action allows the progression of multiple larval-larval molting until the larva attains the required size for metamorphosis [13], [14], [15]. *CYP15C1* is specifically expressed in the corpus allatum (CA), an endocrine organ that produces and secretes JHs. Enzymological analysis revealed that CYP15C1 converts farnesoic acid (FA) to JH acid (JHA) in a highly stereospecific manner. We further demonstrated that CYP15C1 plays an indispensable role in JH biosynthesis, and its molecular defect results in the loss of JHs in the hemolymph, thereby causing precocious metamorphosis in the *mod* strain. Remarkably, precocious larval-pupal transitions in *mod* larvae always occur after the larval third instar, but not in the first or second instar. Our data provide further evidence supporting the hypothesis that authentic (epoxidized) JHs are essential for the classic “status quo” molting in late larval stages (third and fourth instar), but not in early larval stages (first and second instar) of *B. mori* [16].

## Results

### The *mod* strain is a JH–deficient mutant

**Figure 1:**
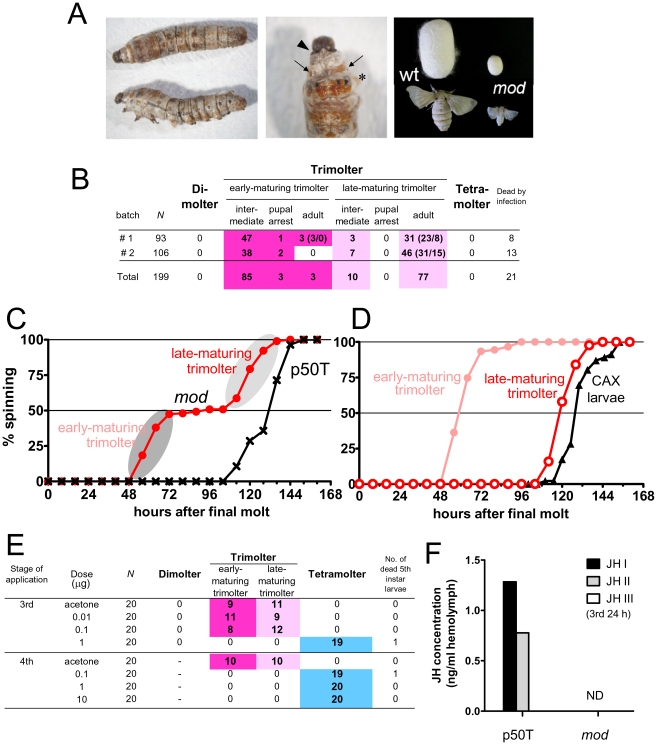
Characterization of the *mod* mutant. (A) Precocious metamorphosis observed in *mod* larvae. (left panel) Lateral and dorsal views and (middle panel) a magnified view of a larval-pupal intermediate. In intermediate animals, the new head capsule of the next instar (fifth) is formed (arrowhead). Beneath the old cuticles (asterisk), a new exoskeleton with larval eye spot markings (arrows) and brown-colored pupal cuticles are formed. (Right panel) Late-maturing trimolters form small cocoons and are able to develop into small but normal adults with normal fertility. (B) The developmental profiles of two batches of *mod* larvae (t011 strain). All of the larvae underwent precocious metamorphosis in the fourth instar, and no dimolters or tetramolters were observed. Larvae could be classified into two groups (early- and late-maturing trimolters) on the basis of the timing of onset of spinning. The numbers in parentheses indicate the sex of the moths (male/female). (C) Timing of the onset of spinning in *mod* (red, n = 178) and p50T (black, n = 28) strains after final larval molting. As highlighted by the grey ellipses, spinning was induced at two distinct timings in the *mod* strain, unlike the p50T strain. (D) Comparison of timings of the onset of spinning among early- and late-maturing trimolters of the *mod* strain and normal strain larvae that had been allatectomized (CAX) at the beginning of the fourth instar. Data on CAX larvae are from [17]; these larvae were reared at relatively low temperatures (23.0–25.5°C), which delays the timing of the onset of spinning to some extent. (E) Methoprene treatment of *mod* larvae. Selected doses of methoprene (0.01–10 µg/larva) were topically applied to newly molted third and fourth instar larvae (8–12 h after molting). As highlighted in blue, precocious pupation could be blocked by methoprene treatment. (F) Measurement of the JH titer in the hemolymph of third instar larvae of p50T and *mod* strains at 24 h after molting. Hemolymph was collected from ∼400 larvae using a microsyringe and the pooled sample was analyzed. JH in the hemolymph was converted to its corresponding methoxyhydrin derivatives and analyzed by GC-MS. JHs were not detected (ND) in the hemolymph of *mod* larvae.

Larvae of standard *B. mori* strains undergo molting four times and thus have five larval instars; these larvae are conventionally termed “tetramolter” in silkworm genetics. The spontaneous mutant *mod* was identified in a standard strain [11] and *mod* larvae undergo precocious metamorphosis in the third (dimolter) or fourth instar (trimolter). First, we obtained a detailed developmental profile of larvae from two batches of the *mod* strain. All *mod* larvae underwent precocious metamorphosis in the fourth instar and no individuals reached the fifth instar (Figure 1A and 1B). We plotted the timing of the onset of spinning in the *mod* larvae (Figure 1C and 1D). Consistent with previous reports [11], [12], we found that spinning occurred at two clearly distinguishable timings: (1) from 56 to 80 h and (2) from 112 to 144 h after the third molt: these larvae were termed early- and late-maturing trimolters, respectively. This segregation in the timing of the onset of spinning was not observed in the standard strain p50T (Figure 1C) or other moltinism strains [11], and thus is a unique characteristic of the *mod* strain. Importantly, development in almost all early-maturing trimolters was arrested and they remained as larval-pupal intermediates (93.4%, 85/91 larvae); only 3 of the 91 larvae (3.3%) successfully survived to adulthood (Figure 1B). In contrast, the late-maturing trimolters did not show such severe developmental impairment and 88.5% (77/87) became miniature adults (Figure 1B). In the larval-pupal intermediates, we usually observed prothetelic phenotypes such as a mixed pupal cuticle on the exoskeleton of animals having overall a larval appearance (Figure 1A), suggesting that hormonal switching of molting and metamorphosis may be aberrant in the *mod* strain. Notably, despite their small body size, reproduction in *mod* moths seemed normal, and their eggs hatched without apparent abnormalities.

In the silkworm, premature metamorphosis can be induced by the loss of or low levels of JH signaling, which can occur due to the surgical removal of the CA [17] or to overexpression of the JH-degrading enzyme [16]. We therefore hypothesized that precocious metamorphosis in the *mod* strain was caused by the prevention of JH biosynthesis or signaling. To examine this hypothesis, we first determined whether the *mod* phenotype could be rescued by treatment with methoprene, a JH analogue. We topically applied several doses of methoprene to newly-molted third or fourth instar *mod* larvae and found that a fourth larval molting was induced by the treatment (Figure 1E). Fifth instar larvae that had undergone fourth larval ecdysis grew normally, began to spin after ∼6 days, and eventually metamorphosed to pupae and adults that were normal and fertile. This result suggests that JH reception and subsequent JH signaling is normal in the *mod* strain. Therefore, we next compared the JH titers in the hemolymph of third instar larvae of *mod* and p50T strains at 24 h after molting to the third instar. JHs were extracted from the hemolymph and their methoxyhydrin derivatives were analyzed by liquid chromatography-mass spectrometry (LC-MS). We detected JH I and JH II in the hemolymph of p50T, whereas the JH titer in the hemolymph of the *mod* strain was below the detectable level (Figure 1F). These results indicate that the *mod* strain is a JH-deficient mutant in which complete (or almost complete) loss of JH caused precocious metamorphosis.

### Positional cloning of the *mod* locus

**Figure 2:**
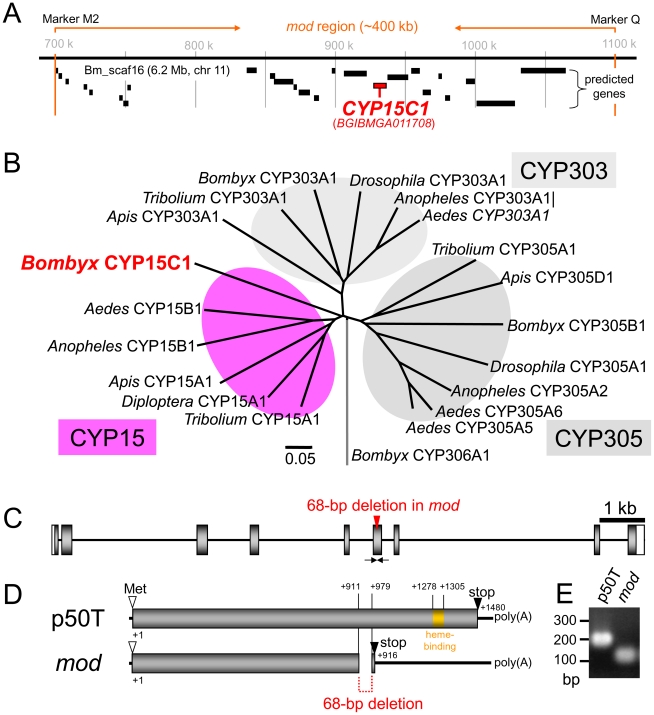
Positional cloning of the *mod* locus. (A) Physical map showing the outcome of the linkage analysis using 792 BC_1_ individuals. The *mod* locus was narrowed to the genomic region flanked by the PCR markers M2 and Q, as indicated by the orange arrows. Putative genes predicted by the Gene model program [8], [9] are shown below the map, and *CYP15C1* (*BGIBMGA011708*) is shown in red. For more details refer to Figure S1. (B) A phylogenetic tree showing the relationship of *CYP15C1* and other related P450 genes. The rootless tree was constructed based on the entire amino acid sequence by the neighbor-joining method using the ClustalX program [55]. Sequences were retrieved from public databases, and the species names are abbreviated as follows: *Aedes*, *A. aegypti*; *Anopheles*, *A. gambiae*; *Apis*, *A. mellifera*; *Bombyx*, *B. mori*; *Diploptera*, *D. punctata*; *Drosophila*, *D. melanogaster*; and *Tribolium*, *T. castaneum*. The scale bar indicates the number of amino acid substitutions per site. Note that CYP15 was not found in *D. melanogaster*. (C) The genomic structure of *CYP15C1* in the wild-type (p50T) strain. White box, grey box, and a black bar indicate untranslated, coding, and intronic regions, respectively. (D) Transcripts of *CYP15C1* from p50T and *mod* strains. A 68-bp deletion was found in *CYP15C1* of the *mod* strain, and this deletion introduced a premature stop codon as indicated in red. Heme-binding motifs of P450s [19] are indicated in orange. (E) Genomic PCR showing the presence of the 68-bp deletion in *CYP15C1* from the *mod* strain. PCR primers (Table S1) that flank the deletion are shown by arrows in (C).

To identify the gene responsible for the *mod* locus, we performed positional cloning using backcross 1 progeny (BC_1_) obtained from crossing females of the *mod* strain (t011 strain, see http://www.shigen.nig.ac.jp/silkwormbase/index.jsp) with F_1_ heterozygote males of *mod* and p50T strains (see Figure S1). We mapped the *mod* locus within ∼400 kb region on the scaffold Bm_scaf16 (chromosome 11) [8] using 792 BC_1_ individuals. Twenty-five genes were predicted to be present within this region. Among them, we focused on *BGIBMGA011708*, a gene encodes a cytochrome P450 monooxygenase. Based on sequence homology and phylogenetic analysis (Figure 2B), the gene was designated as *CYP15C1*. We found that *CYP15C1* shares high homology with the *CYP15A1* of the cockroach *Diploptera punctata*, which is involved in JH biosynthesis in CA of the cockroach [18]. Given that the *mod* phenotype is a result of the loss of the JH titer (Figure 1F), we speculated that the *mod* phenotype is due to the loss of function of *CYP15C1*. To examine this possibility, we first determined the nucleotide sequence of the full-length *CYP15C1* cDNA from p50T and *mod* strains. We identified a 68-bp deletion in the *mod* allele that introduces a premature stop codon in the coding region of *CYP15C1* (Figure 2C–2E). This deletion seemed to produce a functionally null mutation in CYP15C1, since a heme-binding motif, which is essential for enzymatic activities in P450s [19], was eliminated in the *mod* allele (Figure 2D). This result indicates that *CYP15C1* is a strong candidate for the *mod* locus. Therefore, we further characterized *CYP15C1* and its gene product.

### Temporal and spatial expression of *CYP15C1*

**Figure 3:**
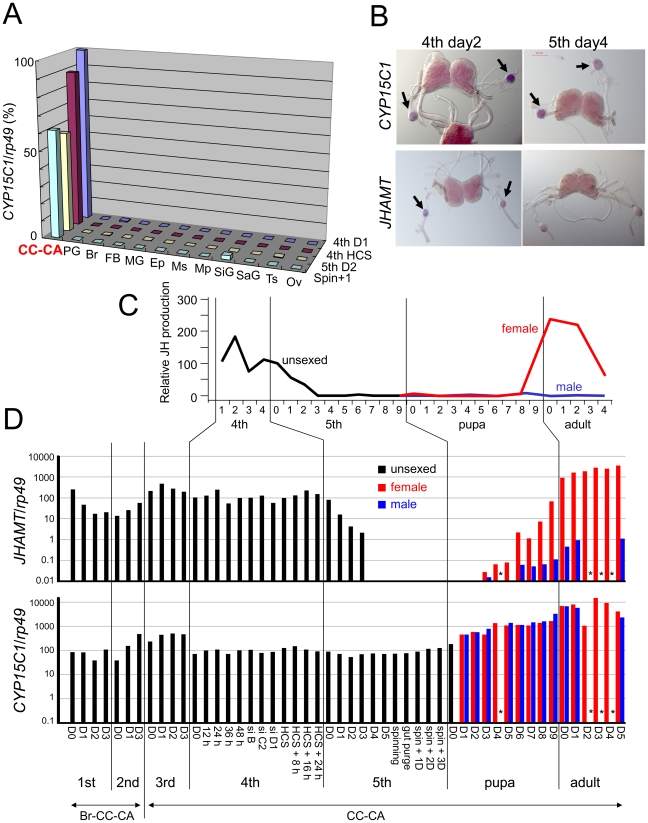
Temporal and spatial expression of *CYP15C1*. (A) qRT-PCR analysis of the spatial expression of *CYP15C1* in the silkworm strain Kinshu×Showa. “*CYP15C1*/*rp49*” on the vertical axis indicates the level of *CYP15C1* mRNA normalized to that of internal *rp49* mRNA. RNAs were collected from larvae on day 1 of the fourth instar (4th D1), fourth instar larvae showing head capsule slippage (4th HCS), larvae on day 2 of the fifth instar (5th D2), and larvae on day 1 after the onset of spinning (Spin+1). CC-CA, corpus cardiacum-corpus allatum complex; PG, prothoracic gland; Br, brain; FB, fat body; MG, midgut; Ep, epidermis; Ms, muscle; Mp, Malpighian tubule; SiG, silk gland; SaG, salivary gland; Ts, testis; and Ov, ovary. (B) *In situ* mRNA hybridization of *CYP15C1* and *JHAMT*. Br-CC-CA complexes on day 2 of the fourth instar and day 4 of the fifth instar were used for analysis. Signals of both genes were limited to CA as indicated by arrows, but *JHAMT* was not detected on day 4 of the fifth instar. The purple coloration in the brain is primarily due to ommochrome pigments and does not reflect gene expression. The result of control experiments using sense probes are shown in Figure S2. (C) Developmental changes in the rate of JH biosynthesis by *B. mori* CA *in vitro*. The data are based on Kinjoh et al. (2007). Black, red, and blue lines indicate CA from unsexed larvae, female and male animals, respectively. The activity in CA on day 1 of the fourth instar was set as 100. (D) Temporal expression patterns of *JHAMT* (upper) and *CYP15C1* (lower) in the Br-CC-CA (first and second instar larvae) or CC-CA (third to fifth instar larvae, pupae, and adults) complex. Developmental stages are defined as h/days after certain developmental events [i.e., molting, head capsule slippage (HCS), spinning, or emergence] or by a spiracle index (si) [56]. Animals were unsexed during larval stages, while sexed during pupal and adult stages (female in red and male in blue). The expression profile of *JHAMT* after the second larval instar is based on published data (20). Expression levels measured on day 2 of the 4th larval instar are arbitrarily set at 100 (for actual transcript numbers per *rp49*) and are shown in a log scale. Asterisks indicate that data were not available.

The strict regulation of JH biosynthesis in CA is critical for the successful development and reproduction of insects [14], [15], [20]. We next examined the spatial expression pattern of *CYP15C1* mRNA. We examined 12 tissues at four different developmental stages and found that *CYP15C1* mRNA was highly specific to the corpus cardiacum (CC)-CA complex (Figure 3A). A whole mount *in situ* hybridization experiment in the brain (Br)-CC-CA complex (Figure 3B and Figure S2) showed that the signal for *CYP15C1* was strictly limited to CA, where JH is synthesized, and could not be detected in the brain or CC. These results showed a close spatial correlation between *CYP15C1* expression and JH biosynthesis.

Next, we carried out a detailed analysis of the temporal expression pattern of *CYP15C1* in the CC-CA complex and compared it to that of the gene for JHA methyltransferase (JHAMT), a key enzyme that acts in the final step of the JH biosynthetic pathway in CA [21]. *CYP15C1* mRNA was constitutively expressed in CA from the first instar larval to adult stages (Figure 3D), even when JH is not synthesized (Figure 3C) [20]; no apparent differences in levels of *CYP15C1* mRNA were observed between males and females during pupal and adult stages (Figure 3D). In contrast, the temporal expression pattern of *JHAMT* correlates well with the JH synthetic activity of CA (Figure 3D and Figure S2). *JHAMT* transcript completely disappeared by day 4 of the fifth instar when CA ceased production of JH (see Figure 3C). It reappeared from the mid-pupal stage and increased to a very high level in the female CA. This was consistent with the temporal profile of JH biosynthesis activity in CA as this occurs only in females during the pupal and adult stages [20]. Taken together, our results strongly indicate that CYP15C1 is involved in JH biosynthesis in CA, but does not appear to act as a rate-limiting factor for JH biosynthesis.

### Enzymatic properties of CYP15C1

**Figure 4:**
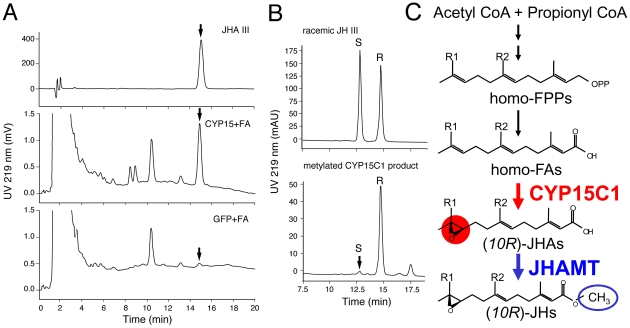
Enzymatic properties of *B. mori* CYP15C1. (A) Enzymatic activity against FA. Medium containing FA was incubated with *Drosophila* S2 cells transiently expressing CYP15C1 (middle) or GFP (bottom), and analyzed by HPLC. Standard JHA III (top). Arrows indicate peaks of JHA III. (B) Stereospecificity. JHA III generated from FA by Sf9 cells stably expressing CYP15C1 (Sf9/CYP15C1) was chemically methylated and analyzed by a Chiral-HPLC. R and S indicate peaks of (*R*)- and (*S*)-JH III enantiomers, respectively. The *R*∶*S* ratio of standard racemic JH III (top) was 50∶50, while that of CYP15C1-produced JH III (bottom) was 97∶3. (C) The late JH biosynthetic step in *B. mori*, in which major JHs in the hemolymph are JH I and II [28]. Ethyl-branched farnesyl diphosphates (homo-FPPs) are converted to homo-FAs, epoxidized to JHAs by the cytochrome P450 epoxidase CYP15C1 (this study), and then methylated by the JHA methyltransferase (JHAMT) [21]. JH I: R1 = R2 = C_2_H_5_, JH II: R1 = C_2_H_5_, R2 = CH_3_.

The cockroach CYP15A1, the ortholog of *B. mori* CYP15C1, catalyzes the epoxidation of (2*E*,6*E*)-methyl farnesoate (MF) to JH III [18]. Although biochemical studies predicted the presence of FA epoxidase in the CA of the lepidopteran insect *Manduca sexta* [22], [23], the corresponding gene has not been identified to date. Therefore we examined the enzymatic activity of *B. mori* CYP15C1 against two plausible substrates, FA and MF. First, we employed a transient expression system using *Drosophila* S2 cells. When S2 cells expressing CYP15C1 were incubated with medium containing FA, a major HPLC peak was generated that had the same retention time (15.1 min) as standard JH III acid (JHA III) (Figure 4A, middle). This peak did not appear when S2 cells expressing GFP were used (Figure 4A, bottom). The ESI-MS spectrum of this peak gave an [M-H]^−^ at *m*/*z* 251, consistent with the C_15_H_24_O_3_ formula of JHA III, confirming that CYP15C1 catalyzes the conversion of FA to JHA III. The enzymatic properties of CYP15C1 was further examined in a stable Sf9 cell line (Sf9/BmCYP15C1) that constitutively expresses CYP15C1. When the Sf9/BmCYP15C1 cells were cultured in medium containing FA, significant levels of JHA III were detected; in contrast, JHA III production was difficult to detect when original Sf9 cells were used (Table S2, Exp.1). When Sf9/BmCYP15C1 cells were cultured in medium containing MF, JH III generation was detected at low levels. However, a similar level of JH III production was also detected in the original Sf9 cells when they were cultured in the same medium (Table S2, Exp.1). These results suggest that JH III production observed in Sf9/BmCYP15C1 was might be due to the presence of endogenous P450 epoxidases in Sf9 cells, which have been reported previously to have lower substrate specificity and stereospecificity [18], [24]. The addition of the JH esterase inhibitor 3-octylthio-1,1,1- trifluropropan-2-one (OTFP) did not increase production of JHA III (Table S2, Exp. 2), indicating that the degradation of JH III by intrinsic JH esterases in the cells was negligible. Therefore, we were able to estimate the conversion ratio of FA and MF to JH III by CYP15C1. This showed that CYP15C1 exhibited at least 18-fold higher activity for FA than MF (Table S2, Exp. 1), a result that is consistent with previous biochemical studies on lepidopteran FA epoxidase in CA.

To further examine the stereospecificity of CYP15C1, the JHA III generated by Sf9/CYP15C1 was chemically methylated and analyzed by a Chiral-HPLC. The methylated product had a major (*R*)-JH III and a minor (*S*)-JH III peak (*R*∶*S* = 97∶3) (Figure 4B). These results show that *B. mori CYP15C1* encodes a functional P450 epoxidase that preferentially converts FA to JHA III rather than MF to JH III, and does so in a highly (*R*)-enantioselective manner (Figure 4C).

### Transgenic rescue experiments using the GAL4/UAS system

**Figure 5:**
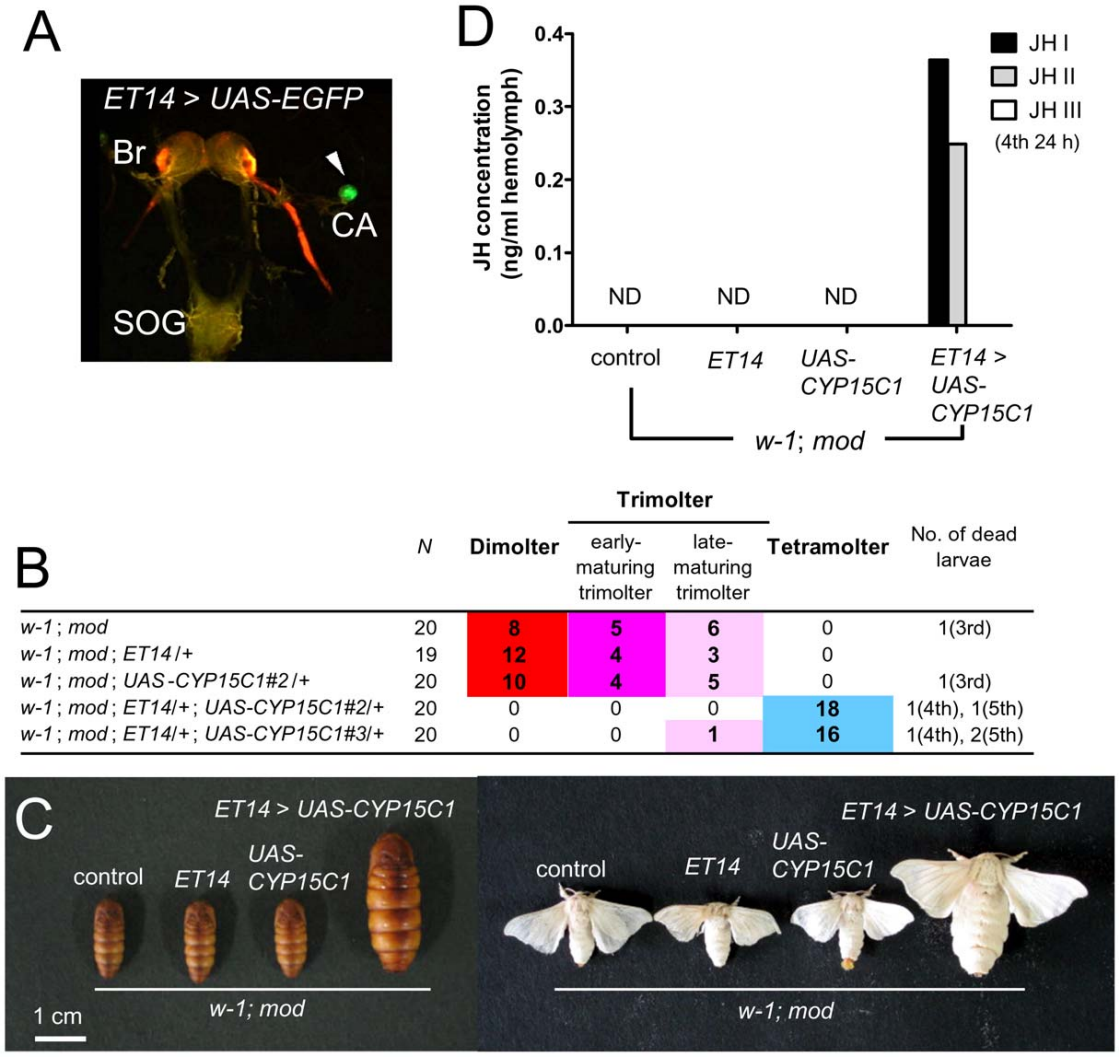
Transgenic rescue of *mod*. (A) Visualization of *GAL4* expression in CA of the enhancer trap line *ET14* carrying the *UAS-GFP* construct. GFP expression (green) is limited to CA (arrowhead). Red fluorescence in the optic nerve is due to DsRed2 expression driven by the 3xP3 promoter [26]. Br, brain; SOG, suboesophageal ganglion; and CA, corpus allatum. (B) Developmental profiles of binary GAL4/UAS transgenic lines. Male moths with a *w-1*; *mod* background and carrying *UAS-CYP15C1* were crossed with *w-1; mod* female moths carrying *ET14*, and their progenies were analyzed. Tetramolters appeared in GAL4/UAS transgenic lines, but not in nonbinary lines. (C) Images of pupae and moths of GAL4/UAS transgenic lines. Larvae carrying both *ET14* and *UAS-CYP151* constructs entered the fifth larval instar and eventually formed larger adults. Control animals did not carry transgenic vectors. (D) Measurement of the JH titer in the hemolymph of GAL4/UAS transgenic lines on the *w-1*; *mod* background. Hemolymph was collected from fourth instar larvae at 24 h after molting and analyzed. JH was detected only in GAL4/UAS lines, but not in nonbinary lines. ND, not detected.

To obtain direct evidence that *CYP15C1* is responsible for the *mod* mutation, we performed transgenic rescue experiments using the GAL4/UAS system [25]. We generated transgenic silkworm lines carrying the *UAS-CYP15C1* transgene with the eye-specific *3xP3-EGFP* marker [26]. The *UAS-CYP15C1* transgene was driven using a silkworm enhancer trap line *ET14* in which *GAL4* was strongly expressed in CA (Figure 5A), although weak expression was also detected in peripheral tissues including fat bodies and the midgut [9], [27]. As these lines were generated using the standard Shiro-C (*w-1*; *+^mod^*) strain, we changed the genetic background to *w-1*/*w-1*; *mod*/*mod* by crossing to the *mod* strain. The resultant *w-1*; *mod*; *ET14*/*+* females were then crossed with *w-1*; *mod*; *UAS-CYP15C1*/*+* males to determine whether the *mod* phenotype could be rescued by *CYP15C1* overexpression. We used two independent *UAS-CYP15C1* lines with *ET-14* (Figure 5B). In both *UAS-CYP15C1* lines, *CYP15C1* overexpression efficiently prevented precocious metamorphosis and 97.1% of the larvae (34/35 in total) underwent the fourth larval molt to become fifth instar larvae (Figure 5B and 5C). Only one larva (1/35) became a late-maturing trimolter, but neither dimolters nor early-maturing trimolters appeared. This result was in contrast to what was observed in control larvae or larvae carrying either the *GAL4* or *UAS* construct alone: approximately half of the larvae became dimolters and the remainder became trimolters, while no larvae became tetramolters. We also measured the JH titer in the hemolymph (Figure 5D). As expected, the JH titers in control, *ET14*, and *UAS* larvae were below the detectable limit. In contrast, we were able to detect JH I and JH II in the hemolymph of *mod* larvae carrying both *ET14* and *UAS-CYP15C1* constructs. Taken together, these results provide direct evidence that *CYP15C1* is responsible for the *mod* mutation and is essential for JH biosynthesis.

## Discussion

In this study, we identified and characterized the gene responsible for the *mod* locus that causes precocious larval-pupal metamorphosis in *B. mori*. The data we present here have two important implications. First, we provide direct genetic evidence for the significance of P450 epoxidase in the late step of the JH biosynthetic pathway, whose expression is essential for normal growth and metamorphosis. Second, we show that the *mod* strain is a JH-deficient mutant strain carrying a null allele of *CYP15C1*, in which developmental abnormalities are mostly limited to larval-pupal transitions and are not observed before the second larval molt.

### Biochemical and physiological function of CYP15C1

**Figure 6:**
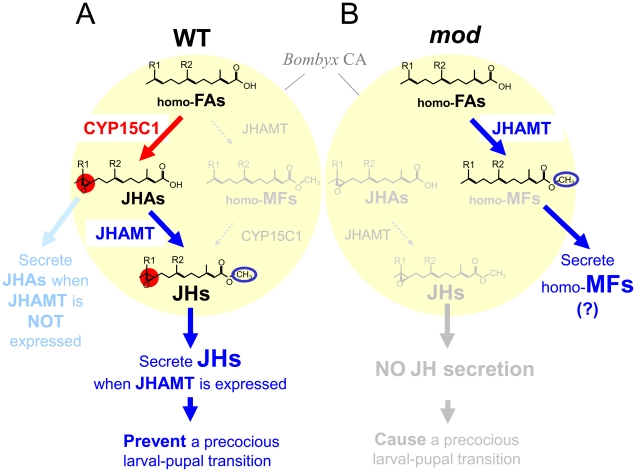
A model for JH biosynthetic pathway in the CA of wt and *mod* silkworms. (A) In the *B. mori* CA, constitutive CYP15C1 expression allows the consistent conversion of homo-FAs to JHAs (predominantly JHA I and II in Lepidoptera). When JHAMT is expressed in CA, JHAs are further converted to JHs, and released from CA, thereby preventing precocious metamorphosis. When JHAMT expression is shut off (e.g., in the prepupal stage), JHAs are likely to be released from CA. (B) In CA of the *mod* strain, homo-FAs are not converted to JHAs because of the loss of CYP15C1, but instead, homo-FAs are converted to ethyl-branched homologs of MF (homo-MFs, i.e., unepoxidized JH I and II) by JHAMT. The loss of CYP15C1 does not allow the conversion of homo-MFs to the authentic JHs. Therefore, neither JHs is synthesized in nor released from CA of the *mod* strain, thereby causing precocious metamorphosis. The synthesized homo-MFs might be released from CA of the *mod* strain, similar to that of higher dipteran insects [57]. JH I: R1 = R2 = C_2_H_5_, JH II: R1 = C_2_H_5_, R2 = CH_3_.

JH III is the most common JH in many insect orders, although its ethyl-branched homologs (JH I and II) are the major JHs in the order Lepidoptera [22], [28]. Biochemical studies have shown that in the late steps of JH biosynthesis in many insect species, including cockroaches and locusts, FA is first methylated to MF and then epoxidized to JH III in CA [22]. However, the final two steps of JH biosynthesis are reversed in Lepidoptera: ethyl-branched homologs of FA (homo-FAs) are first epoxidized and the resultant JHAs (i.e., JHA I and II) are then methylated to the authentic JHs (i.e., JH I and II) [22]. This study showed that *B. mori* CYP15C1 epoxidizes FA to JHA III in a highly stereospecific manner. CYP15C1 might also epoxidize MF to JH III, but in a far less efficient manner (Table S2). Given that *B. mori* JHAMT can methylate both FA and JHAs with similar efficiencies [21], our data clearly demonstrate the major JH biosynthetic pathway in *B. mori*: homo-FAs are first epoxidized to JHAs by CYP15C1, and then methylated to JHs by JHAMT (Figure 4C and Figure 6A). Interestingly, *D. punctata* CYP15A1 does not convert FA to JHA III [18]. Thus, the difference in specificity of CYP15 to the substrates FA and MF may determine the order of the final steps of JH biosynthesis in insects.

The expression of most early JH biosynthetic enzyme genes and *JHAMT* in *B. mori* is limited to the CA and shows dynamic developmental fluctuations [20], [21], [29]. In particular, the temporal expression profile of *JHAMT* correlates well with JH biosynthetic activity in *B. mori* [20], [21], [30], [31] and in the Eri silkworm *Samia cynthia ricini* [32], indicating that *JHAMT* is a key regulatory gene whose transcriptional control is critical for the regulation of JH biosynthesis in Lepidoptera. Here, we found that expression of *CYP15C1* was also limited in CA but in a different pattern to other JH biosynthesis genes in that it was constitutively expressed from larval to adult stages. This result suggests that the transcriptional regulation of CYP15C1 is less important than JHAMT for the temporal regulation of JH production in *B. mori*. CA of the silkworm ceases JH biosynthesis by day 3 of the last (fifth) instar [20]; however, it is speculated that CA synthesizes and secretes JHAs during the following prepupal period. Our data indicate that this endocrine switch can be explained by constitutive *CYP15C1* expression and the shut-off of *JHAMT* expression in CA (Figure 6A). During the larval-pupal transition, homo-FAs are constantly converted to JHA I and II by CYP15C1, and the resultant JHAs are secreted from the gland without further conversion because of the absence of JHAMT.

*CYP15* P450 family members are found in both hemimetabolous and holometabolous insects [33]. In a similar manner as *CYP15C1* expression in *B. mori*, CA-specific *CYP15* expression has also been observed in two cockroach species, *D. punctata* and *Blattella germanica* [18], [34], in the locust *Schistocerca gregaria* [35], and in the mosquito *Aedes aegypti* [36], suggesting a conserved function in JH biosynthesis. However, the enzymatic properties of *CYP15* products, with the exception of those of *D. punctata* [18] and *B. mori* (this study), have not been studied and the physiological role of *CYP15*s in the development of other insects remains unknown. By characterizing the *CYP15C1*-null mutant silkworm, we have demonstrated here that *CYP15C1* plays an essential role in JH biosynthesis and for the maintenance of the proper timing of metamorphosis.

Accumulating data have suggested that *CYP15* genes are evolutionarily diversified in terms of their gene regulation and nature. For example, unlike *B. mori CYP15C1*, *A. aegypti CYP15* shows developmentally and dynamically regulated changes of expression, which appear to correlate well with the JH synthetic activity in the CA [36]. In addition, CYP15 is not present in the genome of *D. melanogaster*, but a P450 gene (*Cyp6g2*) is expressed in CA in a highly tissue-specific manner [37]. More extensive research on the transcriptional controls and enzymatic properties of JH epoxidases across a broader range of insect taxa will shed light on the roles of these enzymes.

### Precocious pupation in *mod* larvae

Our results consistently indicate that the *mod* strain is a JH-deficient mutant that is unable to synthesize JHs in CA. One unique characteristic of the precocious pupation in the *mod* strain is the variation in the timing of the onset of spinning (Figure 1). The feeding period in early-maturing trimolters was unusually short (50 h after molting) compared with that observed in surgical allatectomy of newly molted fourth instar larvae. In the latter larvae, the feeding period was comparable in length to that of the late-maturing trimolters [e.g. ∼130 h [17]] and no timing segregation was observed [17]. In addition, most of the early-maturing trimolters displayed a larval-pupal intermediate phenotype and eventually died, unlike allatectomized larvae, most of which successfully developed into small but normal pupae [17]. One explanation for this phenomenon is that the early-maturing trimolters were destined to undergo larval molting to the fifth instar on day 2, while the late-maturing trimolters were destined for pupation after a prolonged fourth instar, similar to allatectomized larvae [17] (Figure 1D). Molting in early-maturing trimolters on day 2 usually resulted in the formation of larval-pupal intermediates. One possible explanation for this mixed phenotype is that metamorphosis in the *mod* strain is induced in the presence of homo-MFs (unepoxidized JH I and II), presumed products instead of epoxidized JH I and II in CA of the *mod* strain (see Figure 6B). MF is known as a crustacean JH and has recently been reported to have JH activity in *D. melanogaster* [38], [39]. Therefore, MF and its homologs might have JH-like activity but not able to fully substitute for authentic (epoxidized) JHs in the physiology of the silkworm. Alternatively, other P450 epoxidases in *B. mori* that have low substrate specificity and stereospecificity, like CYP9E1 [18] and CYP6A1 [24] in other insects, might substitute for the absence of CYP15C1 in peripheral tissues of *mod* larvae, and such locally-synthesized JHs may prevent precocious metamorphosis in the first and second instar larvae carrying the *mod* mutation. Further studies are needed to elucidate the mechanism for this unique characteristic of the *mod* strain.

We found that the precocious phenotype was more severe in the *w-1*; *mod* strain compared to that in t011, a genetic stock of the *mod* strain. We rarely observed dimolter larvae in the t011 stock (Figure 1B). However, in the original manuscript in 1956, it was reported that 28–92% of *mod* larvae became dimolters [11]. This difference might have developed as a consequence of unintended artificial selection during stock maintenance that favored broods producing trimolters in higher proportions, as it is difficult to obtain sufficient number of eggs using dimolter moths [11], [12]. Thus, we speculate that the present t011 stock may be genetically fixed to produce mostly trimolters, and that this attribute can be varied by outcrossing to other strains.

In the silkworm, premature metamorphosis can be induced by surgical removal of JH-producing CA (allatectomy) [17], by application of an imidazole-based insect growth regulator KK-42 [40] or an anti-juvenile hormone agent KF-13S [41], [42], or by continuous overexpression of the JH-degrading enzyme, JH esterase [16]. In any case, however, premature pupation is not induced in larvae younger than the third instar. In agreement with these studies, we did not observe precocious pupation in first or second instar *mod* larvae, nor did we observe apparent developmental abnormalities during these early instars. Therefore, our data support the hypothesis that there are two physiological phases in the life of silkworm larvae [16]: the JH-independent phase (first and second instar) in which JH does not have a morphogenetic function; and, the JH-dependent phase (third instar and thereafter) in which the morphostatic action of JH is required to prolong the larval stage until the attainment of the appropriate body size for metamorphosis. Given that most generally the minimum number of the larval instar in insects is three [1], [2], our data further imply that insect larvae need to experience at least one [e.g., L2 pupae in *D. melanogaster* [43]] or two (e.g., *B. mori*) larval-larval molts and/or require a certain length of time of postembryonic development in order to acquire competence for metamorphosis.

The silkworm is a classic model organism that has been used for pioneering studies in genetics, physiology, and biochemistry [5]. The availability of whole genome data [8], post-genomic tools [10], and unique mutant resources [6], together with the classic “status quo” responses to JHs in this insect [14], [15], [17], makes the silkworm well-suited for study of hormonal control of growth and development. Indeed, these advantages have greatly contributed to the identification of essential components in the biosynthesis of ecdysteroids, the insect molting hormones [44]. Moreover, recent success in targeted gene disruption using a zinc-finger nuclease [45] increases the utility of this model organism. We are hopeful that our present study will encourage further studies on other “moltinism” strains in the silkworm, and consequently pave the way for a greater understanding of physiological control, developmental plasticity, and evolutionary history of the number of larval molting in insects, which may reflect adaptive strategies of insects to diverse environmental conditions. It is also noteworthy that the late step of the JH biosynthetic pathway is insect-specific and is therefore a potential target for biorational insecticides [46].

## Materials and Methods

### Insects and cell lines

Silkworms were reared on an artificial diet or mulberry leaves at 25–27°C under standard conditions as described previously [47]. The silkworm strain t011 (*mod*/*mod*) was obtained from Kyushu University [6]. The *Spodoptera frugiperda* Sf9 and *Drosophila melanogaster* S2 cells were maintained as described previously [48]. To determine the developmental profile of *mod*, larvae from two batches of t011 were individually reared in plastic dishes, and their developmental stages were recorded at ∼8-h intervals.

### Hormonal treatments

The JH analog, methoprene (a kind gift from S. Sakurai) was applied to newly molted third or fourth instar larvae (∼8–12 h after molting). Methoprene was diluted with acetone and the selected doses (0.01–10 µg/larva) were topically applied to the dorsum using a 10-µl Hamilton microsyringe. The same volume of acetone was applied as a control.

### Positional cloning of the *mod* locus

Positional cloning of the *mod* locus was performed as described previously [49]. Codominant PCR markers and p50T-specific PCR markers were generated for each position of the scaffold Bm_scaf16 (chromosome 11) [9], and used for genetic analysis (Figure 2A and Figure S1). Homozygotes of the *mod* locus were collected from the BC_1_ population [t011×(p50×t011)] based on the phenotype of precocious pupation.

### Cloning of *CYP15C1*

Total RNAs were collected from CA of day 0 fifth instar larvae of p50T and Kinshu×Showa strains and used for 5′- and 3′-rapid amplification of cDNA ends (RACE) using the GeneRacer Kit (Invitrogen). PCR was performed using the primers listed in Table S1. The PCR products were subcloned and sequenced as described previously [47]. The obtained cDNA sequence was deposited in the GeneBank (accession number: AB124839).

### Quantitative RT–PCR (qRT–PCR) analysis

qRT-PCR was performed essentially as described previously [21]. The primers used for the quantification of the *CYP15C1* transcript are listed in Table S1.

### *In situ* hybridization

*In situ* hybridization was performed essentially as described previously [50]. A *CYP15C1* cDNA fragment (∼1.1 kb) was amplified by PCR listed in Table S1 and subcloned into a pDrive plasmid vector (QIAGEN).

### Chemicals

(2*E*,6*E*)-farnesoic acid (FA) and (2*E*,6*E*)-methyl farnesoate (MF) were purchased from Echelon Research Laboratories (Salt Lake City) and racemic JH III from Sigma. JH III acid was prepared from the racemic JH III as described previously [21]. (*R*)-JH III was a kind gift from W.G. Goodman.

### Enzyme assays of CYP15C1 in S2 cells

*CYP15C1* overexpression in S2 cells was achieved using a GAL4/UAS system [51]. To generate a vector for expressing *CYP15C1* under the control of the *UAS* promoter (*UAS-CYP15C1-HA*), a cDNA fragment coding the entire *CYP15C1* ORF was ligated into the pUAST vector. *UAS-GFP.RN3* [52] was used as a negative control. *UAS-CYP15C1-HA* or *UAS-GFP.RN3* was transfected with the *Actin5C-GAL4* construct (a gift from Yasushi Hiromi, National Institute of Genetics, Japan). Forty-eight hours after transfection of S2 cells in a 60-mm dish, the old medium was replaced with 2 ml of fresh medium. S2 cells were detached from the bottom of the dish by pipetting, and 1 ml of the cell suspension was transferred to a siliconized glass test tube. FA or MF (100 µM at final concentration) was then added to the tube. After incubation at 25°C for 16 h, 500 µl of medium was collected and mixed with 500 µl of acetonitrile. Samples were centrifuged for 10 min at 15,000 rpm, followed by incubation at 25°C for 10 min. After filtration using a 0.2 µm filter, 10–20 µl of each sample was subjected to HPLC analysis as described below.

### Establishment of Sf9 cells stably expressing CYP15C1 and enzyme assay

A cDNA with the full ORF of *CYP15C1* cDNA was subcloned into the pIZT/V5-His vector (Invitrogen). The plasmid was transfected into Sf9 cells with Cellfectin reagent (Invitrogen), then cells transiently expressing *CYP15C1* were selected successively with Zeocin according to the manufacture's instruction and a cell line (Sf9/CYP15C1) stably expressing *CYP15C1* was established. Sf9/CYP15C1 cells were placed in a glass tube (12×75 mm) coated with PEG20,000 and cultured in SF900-II SFM medium containing FA or MF (10 µg/ml) for either 2 or 6 h at 26°C. In some experiments, the specific JH esterase-specific inhibitor OTFP (6 µM) was added to the medium to prevent possible degradation of the generated JH III by intrinsic JH esterase present in the cells. After incubation, an equal volume of CH_3_CN was added to the medium, vortexed vigorously and centrifuged for 4,800 rpm for 10 min to remove cell debris. The supernatant was directly subjected to an HPLC analysis as described below for JH III acid or JH III, which were expected to be generated from FA and MF, respectively.

### HPLC and ESI-MS analyses of JH III and JH III acid

JH III was analyzed by reversed-phase HPLC as described previously [21]. JH III acid was analyzed by reversed-phase HPLC (column, Shiseido ODS UG80, 150 mm×3.0 mm ID; solvent, CH_3_CN-20 mM CH_3_COONH_4_, pH 5.5, 35∶65, flow rate, 0.5 ml/min; detection, UV 219 nm). ESI-MS spectrum of JH III acid was obtained by TSQ system (Thermo Quest Finnigan, USA).

### Analysis of the stereospecificity of JH III acid generated by CYP15C1

The stereospecificity of the epoxide group of JH III acid formed by CYP15C1 was analyzed as follows under semi-dark conditions. Sf9/CYP15 cells were cultured in medium containing 10 µg/ml FA for 48 hrs. An equal volume of CH_3_CN was added to the medium (2 ml), vortexed vigorously and centrifuged at 4,800 rpm for 10 min. One ml of 1 M CH_3_COONH, (pH 5.5) was added to the supernatant and extracted with 5 ml of CH_2_CH_2_; this step was performed 5 times. The extract was dehydrated with anhydrous Na_2_SO_4_ and evaporated to dryness in vacuo at 40°C, then the residue was dissolved in 200 µl of CH_2_Cl_2_, 50 µl of MeOH and 100 µl of TMS-diazomethane were then added and the solution was incubated at room temperature for 30 min. The reaction was dried with an N_2_ gas stream, the residue dissolved in 100 µl of hexane, and subjected to a normal-phase HPLC (column, Shiseido SG80, 250×4.6 mm ID; solvent, hexane-EtOH, 99∶1; flow rate, 0.5 ml/min; detection, UV 211 nm). The peak corresponding to JH III (r.t. = 9.8 min) was collected. The stereospecificity of the epoxide group of the JH III was analyzed by a chiral-HPLC (column, Chiralapack IA, 250×4.6 mm ID, DAICEL; solvent, hexane-EtOH, 99∶1; flow rate, 0.5 ml/min; detection UV 219 nm) as described previously [31].

### Purification of JHs from hemolymphs and preparation for LC-MS analysis

Ten microliters of deuterium-substituted JH III (d_3_-JH III) [53] in toluene (67.1 pg/ml) was transferred to a clean glass tube to which 0.5 ml of methanol was added. The hemolymph sample (100 µl) was then added and mixed vigorously, and 1.5 ml of 2% NaCl was added to the JH sample. JH was extracted by partition with 0.5 ml hexane; this step was performed three times. The combined solvent containing JH (1.5 ml) was evaporated under a stream of nitrogen. One hundred microliters of methanol and 2 µl trifluoroacetic acid were added to the crude JH extract and mixture heated at 60°C for 30 min. After removal of the methanol, methoxyhydrin derivatives of JH (JH-MHs) were purified using a Pasture pipette packed with 1.0 g of aluminum oxide (activity grade III, ICN Ecochrom) prewashed with hexane. After loading the extract and washing with 2 ml of 30% ether in hexane, JH-MHs were eluted with 2 ml of 50% ethyl acetate in hexane and then dried under a stream of nitrogen. The residue was dissolved in 25 ml of 80% acetonitrile containing 5 µM sodium acetate.

### Analytical condition for LC-MS

The HP1100MSD system (Agilent) was equipped with a 150×3 mm C18 reversed phase column (UG80, Shiseido) protected by a guard column with 70% acetonitrile containing 5 µM sodium acetate at a flow rate of 0.4 ml/min. For MS analysis, electrospray ionization in the positive mode was used under the conditions of drying gas temperature at 320°C with 10 l/min flow rate, ionization voltage of 70 V. Under these conditions, selected ion masses for each JH-MH were monitored as [M+Na]+, i.e., m/z 321, 324, 335, and 349 for JH III, d_3_-JH III, JH II, and JH I, respectively.

### Transgenic rescue experiments

Overexpression of *CYP15C1* was performed in transgenic silkworms using the GAL4/UAS system as described previously [25], [27], [54]. A coding sequence of CYP15C1 was introduced into a silkworm UAS vector carrying the marker gene *3xP3*-*EGFP*. *B. mori* transformants were established using standard protocols [10]. To overexpress *CYP15C1* on the *mod*/*mod* background, established UAS lines and an enhancer trap line *ET14* [27] were crossed with the t011 strain, and the resultant F_1_ animals were sib mated to obtain the F_2_ generation. In the F_2_ generation, we collected animals showing premature pupation with white eyes (i.e., *mod*/*mod*; *w-1*/*w-1*) and confirmed the presence of the fluorescent marker gene using a fluorescent microscope (SZX12, Olympus). The established *w-1*; *mod* lines carrying *UAS-CYP15C1* or *ET14* were crossed, and their offspring were examined to determine whether precocious metamorphosis was blocked by *CYP15C1* overexpression.

## Supporting Information

Figure S1 Detailed procedure for positional cloning of the *mod* locus. (A) Mating scheme for mapping the *mod* locus. A single-pair cross between a female p50T (wt) and a male t011 (*mod*/*mod*) [6] produced the F_1_ offspring. Then, the male informative cross (t011 female×F_1_ male) produced the BC_1_ progeny. We collected and analyzed 792 BC_1_ individuals with the *mod* phenotype (premature pupation). (B) The result of fine mapping of the *mod* locus. We generated 12 PCR markers for each position of the scaffold Bm_scaf16 [9] that showed polymorphisms between p50T and t011 strains. We analyzed 792 BC_1_ individuals and the results are summarized in the Table. “m” indicates the t011/t011 homozygous genotype, “p/m” indicates the p50T/t011 heterozygous genotype, and “p” indicates the genotype carrying a p50T-specific allele in each marker. The genomic region for the *mod* locus lies between the Q and M2 markers, as indicated by red arrows. (C) PCR markers used in this study.
(DOC)

Figure S2 Whole-mount *in situ* hybridization in the brain-CC-CA complex. Whole-mount *in situ* hybridization of *CYP15C1* and *JHAMT* in the brain-CC-CA complex on day 2 of the fourth instar and day 4 of the fifth instar. Magnified images of CAs indicated by arrows are shown below each panel. Signals were not detected when sense probes were used for analysis.
(DOC)

Table S1 PCR primers used in this study.
(DOC)

Table S2 Substrate specificity of CYP15C1 to FA and MF. Sf9 or Sf9/CYP15C1 cells (∼1.2×10^6^) were cultured with 200 ml of medium containing 2 µg of FA or MF at 26°C for 2 h (Exp. 1) or 6 h (Exp. 2), and the production of JHA III or JH III in the medium was quantified by HPLC. Mean ± SD (N = 3). ND, not detected.
(DOC)
