# Supplementary figures and images for: Precocious Metamorphosis in the Juvenile Hormone–Deficient Mutant of the Silkworm, Bombyx mori
Source: PLoS Genet. 2012 Mar 8;8(3):e1002486. doi: 10.1371/journal.pgen.1002486 (PMC3297569; doi:10.1371/journal.pgen.1002486)

Figure S1.


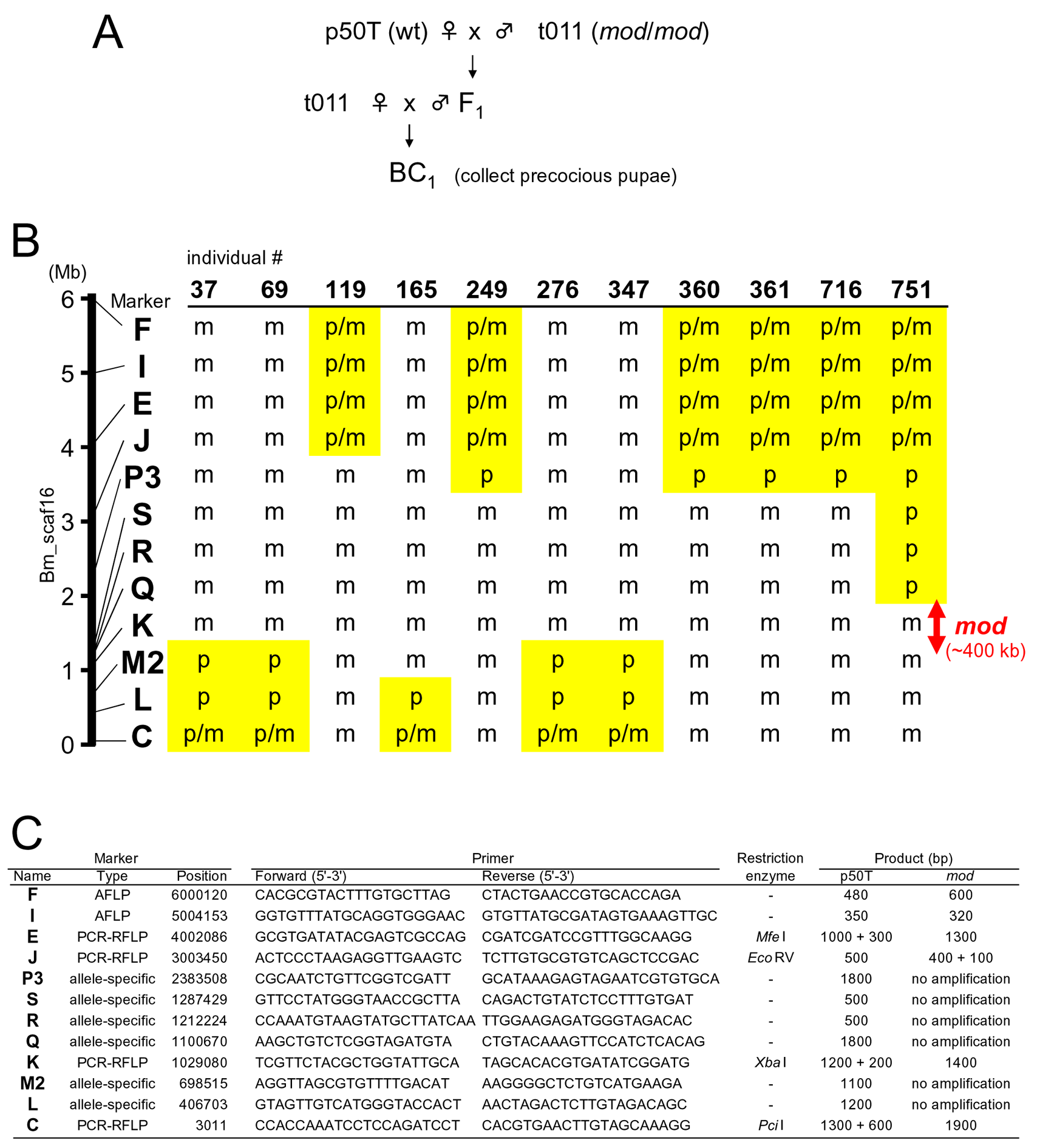

Supplement: Figure S1 — Detailed procedure for positional cloning of the mod locus. (A) Mating scheme for mapping the mod locus. A single-pair cross between a female p50T (wt) and a male t011 (mod/mod) [6] produced the F1 offspring. Then, the male informative cross (t011 female×F1 male) produced the BC1 progeny. We collected and analyzed 792 BC1 individuals with the mod phenotype (premature pupation). (B) The result of fine mapping of the mod locus. We generated 12 PCR markers for each position of the scaffold Bm_scaf16 [9] that showed polymorphisms between p50T and t011 strains. We analyzed 792 BC1 individuals and the results are summarized in the Table. “m” indicates the t011/t011 homozygous genotype, “p/m” indicates the p50T/t011 heterozygous genotype, and “p” indicates the genotype carrying a p50T-specific allele in each marker. The genomic region for the mod locus lies between the Q and M2 markers, as indicated by red arrows. (C) PCR markers used in this study. (DOC) [file pgen.1002486.s001.doc]

Figure S2.


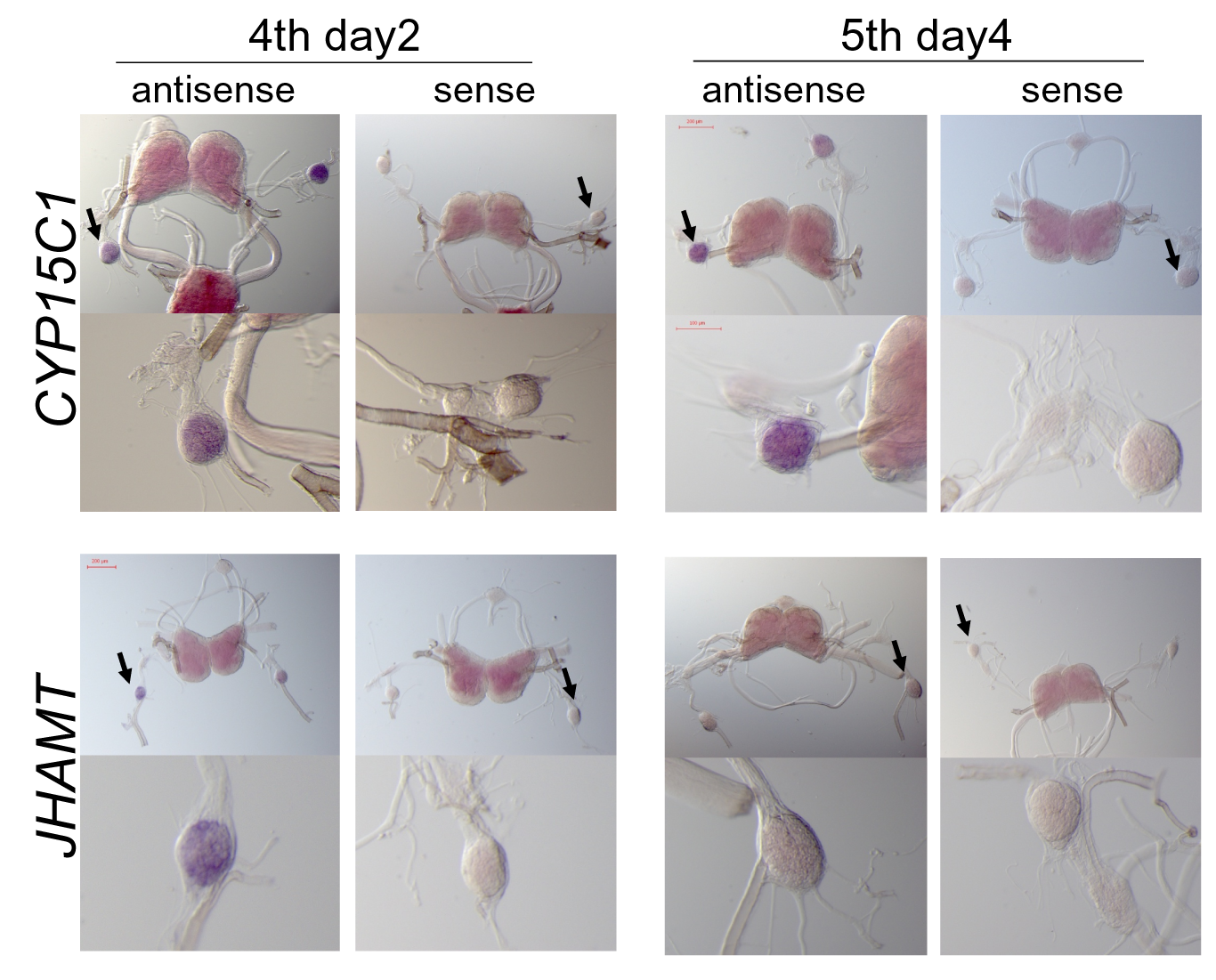

Supplement: Figure S2 — Whole-mount in situ hybridization in the brain-CC-CA complex. Whole-mount in situ hybridization of CYP15C1 and JHAMT in the brain-CC-CA complex on day 2 of the fourth instar and day 4 of the fifth instar. Magnified images of CAs indicated by arrows are shown below each panel. Signals were not detected when sense probes were used for analysis. (DOC) [file pgen.1002486.s002.doc]
