# Supplementary material for: Precocious Metamorphosis in the Juvenile Hormone–Deficient Mutant of the Silkworm, Bombyx mori
Source: PLoS Genet. 2012 Mar 8;8(3):e1002486. doi: 10.1371/journal.pgen.1002486 (PMC3297569; doi:10.1371/journal.pgen.1002486)
Supplement: Table S1 — PCR primers used in this study. (DOC) [file pgen.1002486.s003.doc]

| Table S1. PCR primers used in this study. | |  |
| --- | --- | --- |
|  |  |  |
| **Name** | **Sequence (5'- to -3')** | **Purpose** |
| RACE-GSP-F1 | TTGATCTACTTGAAGCGGGCATGGAGACAG | 5'-RACE of *CYP15C1* |
| RACE-GSP-R1 | GCCCATAGATGCGACAGTCGATATCCTCA | 3'-RACE of *CYP15C1* |
| CYP15ORF-F1 | CCTAGGATGTTGGCATTAATTGCTCTT | Construction of a UAS vector for transgenesis |
| CYP15ORF-R1 | CCTAGGAACGTGTTTATGTGGACAAATG | Construction of a UAS vector for transgenesis |
| CYP15gPCR-F1 | CAACGCTTGATGATCTACAAGTA | Detection of 68-bp deletion in *CYP15C1* of *mod* |
| CYP15gPCR-R1 | CTTATTCTGTCGTCAAGCAAAG | Detection of 68-bp deletion in *CYP15C1* of *mod* |
| CA23j-5 | GAATTCATGTTGGCATTAATTGTTC | Construction of a UAS vector for enzyme assays in S2 cells |
| CA23j-3 | GCGGCCGCCAATACTCTTAAATGTTTTTC | Construction of a UAS vector for enzyme assays in S2 cells |
| qRT-CYP15-F1 | CATCACGGACCACATTGGAAAG | qRT-PCR of *CYP15C1* |
| qRT-CYP15-R1 | CAAAAGGTATGAGCCACTCGTCTTG | qRT-PCR of *CYP15C1* |
| insituCYP15-F1 | TCATAAGCCGACATC | Synthesis of *CYP15C1* probe for *in situ* hybridyzation |
| insituCYP15-R1 | TCCAATGTGGTCCGTGAT | Synthesis of *CYP15C1* probe for *in situ* hybridyzation |
|  |  |  |
