# Supplementary material for: Precocious Metamorphosis in the Juvenile Hormone–Deficient Mutant of the Silkworm, Bombyx mori
Source: PLoS Genet. 2012 Mar 8;8(3):e1002486. doi: 10.1371/journal.pgen.1002486 (PMC3297569; doi:10.1371/journal.pgen.1002486)
Supplement: Table S2 — Substrate specificity of CYP15C1 to FA and MF. Sf9 or Sf9/CYP15C1 cells (∼1.2×10^6^) were cultured with 200 ml of medium containing 2 µg of FA or MF at 26°C for 2 h (Exp. 1) or 6 h (Exp. 2), and the production of JHA III or JH III in the medium was quantified by HPLC. Mean ± SD (N = 3). ND, not detected. (DOC) [file pgen.1002486.s004.doc]

| Table S2. Substrate specificity of CYP15C1 to FA and MF | | | | |  |  |
| --- | --- | --- | --- | --- | --- | --- |
|  |  |  |  |  | Product | |
|  | Cell | Substrate (2 μg) | OTFP | Culture time (h) | JHA III | JH III |
| Exp. 1 | Sf9 | FA | - | 2 | ND | - |
|  | Sf9 | MF | - | 2 | - | 9.3 ± 1.4 |
|  | Sf9/CYP15C1 | FA | - | 2 | 402 ± 9.7 | - |
|  | Sf9/CYP15C1 | MF | - | 2 | - | 22.3 ± 3.8 |
| Exp. 2 | Sf9/CYP15C1 | MF | - | 6 | - | 118.7 ± 13.3 |
|  | Sf9/CYP15C1 | MF | 6 μM | 6 | - | 73.3 ± 26.6 |
| Sf9 or Sf9/CYP15C1 cells (~1.2 x 10^6^) were cultured with 200 ml of medium containing 2 μg of FA or MF at 26 ^o^C for 2 h (Exp. 1) or 6 h (Exp. 2), and the production of JHA III or JH III in the medium was quantified by HPLC. Mean ± SD (N = 3). ND, not detected. | | | | | | |
|  |  |  |  |  |  |  |
|  |  |  |  |  |  |  |
|  |  |  |  |  |  |  |
|  |  |  |  |  |  |  |
